# In Vitro and In Vivo Nephroprotective Effects of *Nelumbo nucifera* Seedpod Extract against Cisplatin-Induced Renal Injury

**DOI:** 10.3390/plants11233357

**Published:** 2022-12-02

**Authors:** Jui-Yi Chen, Chia-Lin Tsai, Chiao-Yun Tseng, Pei-Rong Yu, Yu-Hsuan Chang, Yue-Ching Wong, Hui-Hsuan Lin, Jing-Hsien Chen

**Affiliations:** 1 Division of Nephrology, Department of Internal Medicine, Chi Mei Medical Center, Tainan City 71004, Taiwan; 2 Department of Health and Nutrition, Chia Nan University of Pharmacy and Science, Tainan City 71710, Taiwan; 3 Department of Nutrition, Chung Shan Medical University, Taichung City 40201, Taiwan; 4 Department of Medical Laboratory and Biotechnology, Chung Shan Medical University, Taichung City 40201, Taiwan; 5 Department of Medical Research, Chung Shan Medical University Hospital, Taichung City 40201, Taiwan

**Keywords:** cisplatin, lotus seedpod extract, nephrotoxicity, apoptosis

## Abstract

Cisplatin has been considered a chemotherapeutic drug for treating human tumors, and one of the noteworthy side effects of cisplatin is nephrotoxicity. Amelioration of cisplatin-induced nephrotoxicity is necessary. Lotus seedpod extract (LSE) mainly composed of quercetin-3-glucuronide has been revealed for antioxidant and anti-tumor effects. However, the effects of LSE on cisplatin-induced nephrotoxicity are still unknown. This study aims to explore the in vitro and in vivo protective effect and possible mechanism of LSE on cisplatin-induced nephrotoxicity. Results showed that co-treatment of LSE with cisplatin raised the viability of rat renal tubular epithelial NRK−52E cells and decreased oxidative stress and cell apoptosis when compared to the cells treated with cisplatin alone. The molecular mechanisms analyzed found that LSE could reduce the expressions of apoptotic factors, including Bax, Bad, t-Bid, and caspases. In the in vivo study, LSE improved the cisplatin-induced levels of serum markers of kidney function, glomerular atrophy, and the degree of apoptosis in the kidneys. This is the first study to display that LSE prevents cisplatin-induced nephrotoxicity by reducing oxidative stress and apoptosis. Thus, LSE could be a novel and natural chemoprotective agent for cisplatin chemotherapy in the future.

## 1. Introduction

Cisplatin has commonly been used for the treatment of cancers such as ovarian cancer and head and neck cancer. Among the side effects of cisplatin treatment is known nephrotoxicity. Cisplatin is transferred into renal epithelial cells through copper transporters and organic cation transporters [1]. The mechanism of cisplatin cytotoxicity is mediated by the interaction of cisplatin with DNA [2]. Cisplatin could alter the secondary structure of DNA, inhibiting the template function and replication [3,4]. When it comes to DNA damage, the p53 tumor suppressor is phosphorylated to regulate cell cycle arrest and apoptosis in response to DNA damage [5]. In addition, cisplatin causes mitochondrial DNA damage resulting in an accumulation of reactive oxidative species (ROS) [6]. The p53 tumor suppressor and ROS lead to mitochondrial dysfunction, which releases cytochrome C and increases the activity of caspase 9 to direct the cell to an apoptotic pathway [7].

Apoptosis, commonly known as programmed cell death, is initiated through different pathways including the cell-surface death receptor (extrinsic) pathway and mitochondrion-initiated (intrinsic) pathway [8]. The extrinsic pathway is triggered by receptors such as Fas and TNFR_1_ at the surface of cells [9]. When DNA is damaged, the p53 tumor suppressor is phosphorylated, and the intrinsic pathway is activated by Bcl-2 family proteins that could govern mitochondrial membrane permeability [10]. Extrinsic and intrinsic pathways involve procaspase 8 and procaspase 9 interactions, respectively. Both caspase 8 and caspase 9 lead to the activation of caspase 3, which plays an important role in apoptosis and executes the final phase of apoptotic cell death [11].

Lotus (*Nelumbo nucifera*) is a perennial aquatic herb that is mostly planted in the tropics such as in Taiwan. Most lotus was considered as traditional medicine in Asia. The lotus seedpod is the receptacle of lotus and has been considered as waste after the harvest [12]. Previous studies showed that lotus seedpod was rich in flavonoid compounds such as quercetin-3-glucuronide (Q3G), isorhamnetin-3-glucuronide, and isorhamnetin-3-glucoside [13]. Lotus seedpod extract was reported to possess anti-tumor effects, inhibit advanced glycation end-product formation [14], ameliorate cognitive impairment [15], restrain the inflammatory response [16], and improve the oxidative stress in obese mice [17]. Based on the above results, LSE could be applied to disease prevention via the improvement in oxidative stress. However, there was no relevant research about the protective effects of lotus seedpod against cisplatin-induced renal toxicity.

This study aims to investigate the nephroprotective effects and mechanism of LSE and the hypothesis in this research was that LSE reduces cisplatin-induced oxidative stress to improve nephrotoxicity. In the in vitro study, NRK−52E cells were used under the induction of cisplatin to analyze ROS production and the expression of apoptosis-related proteins. In the in vivo study, LSE was applied topically to a nude mice model under cisplatin treatment to observe the histological section and antioxidant capacity and finally confirm the nephroprotective effects of LSE.

## 2. Results

### 2.1. Effect of LSE on Cell Viability in Cisplatin-Induced NRK-52E Cells

**Figure 1:**
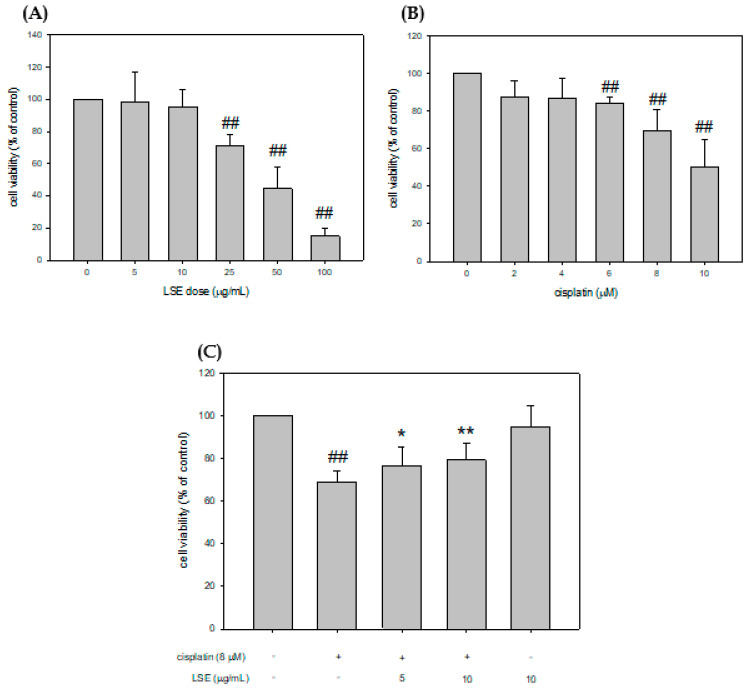
Effect of LSE on the survival of NRK−52E cells and effect of cisplatin with/without LSE on NRK-52E cell viability. (**A**) NRK−52E cells were treated with 0, 5, 10, 25, 50, and 100 μg/mL of LSE for 24 h. Cellular survival was measured by Guava^®^ Muse^®^ cell analyzer. (**B**) NRK−52E cells were treated with 0, 2, 4, 6, 8, and 10 μM of cisplatin for 24 h. Cell survival was measured by Guava^®^ Muse^®^ cell analyzer. (**C**) NRK−52E cells were treated with or without LSE (5, 10 μg/mL) for 24 h in the presence or absence of cisplatin (8 μM). Quantification data were presented as mean ± SD of three independent experiments by using Sigma plot 10.0 software. ## *p* < 0.01 compared with the control group; * *p* < 0.05, ** *p* < 0.01 compared with the cisplatin group.

This study examined the effect of LSE on the cell viability of cisplatin-induced NRK−52E cells in different concentrations for 24 h treatment to investigate the survival rate after LSE intervention. Cell viability results indicated that LSE in 5 and 10 μg/mL concentrations did not affect the survival rate of NRK−52E cells (Figure 1A). The survival rates of cells treated with cisplatin in different concentrations (2, 4, 6, 8, and 10 μM) were tested, and the results revealed that cisplatin did had no effect under 6 μM, whereas a higher dose of cisplatin (8 and 10 μM) inhibited the cell survival rate (Figure 1B). Based on these results, LSE concentration of 5 and 10 μg/mL and a cisplatin concentration of 8 μM were chosen for further experiments. To understand whether the LSE intervention could improve NRK−52E cell damage caused by cisplatin, LSE in 5 and 10 μg/mL concentrations were co-treated with 8 μM cisplatin. Finally, the cell survival rate was significantly raised by 10.6% and 14.4% for the different LSE concentrations (5 and 10 μg/mL) compared with the cisplatin group after cotreatment for 24 h. These results indicate that LSE intervention could ameliorate cisplatin-induced cell death.

### 2.2. Effect of LSE on Cisplatin-Induced ROS Production in NRK−52E Cells

**Figure 2:**
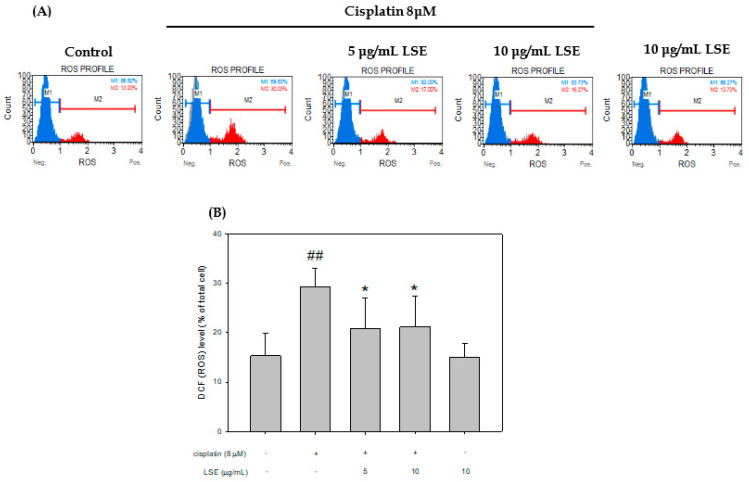
Effect of LSE on cisplatin-induced ROS production in NRK−52E cells. NRK−52E cells were treated with or without LSE (5, 10 μg/mL) for 24 h in the presence or absence of cisplatin (8 μM). (**A**) The ROS production was assayed with flow cytometry. (**B**) The DCF-positive cells divided by the total number of cells were calculated. Quantification data were presented as mean ± SD of three independent experiments by using Sigma plot 10.0 software. ## *p* < 0.01 compared with the control group; * *p* < 0.05 compared with the cisplatin group.

The ROS level of cisplatin-induced NRK−52E cells with/without LSE treatment was analyzed, and the data were displayed in Figure 2. The ROS production level of the cisplatin group was significantly higher than the control group by 14%. After cotreatment with LSE (5 and 10 μg/mL) and cisplatin, the ROS level was significantly decreased by 8.43% and 8.14% in the cisplatin group. These results show that LSE could reduce the ROS level in cisplatin-induced NRK−52E cells.

### 2.3. Effect of LSE on Cisplatin-Induced Apoptosis in NRK−52E Cells

**Figure 3:**
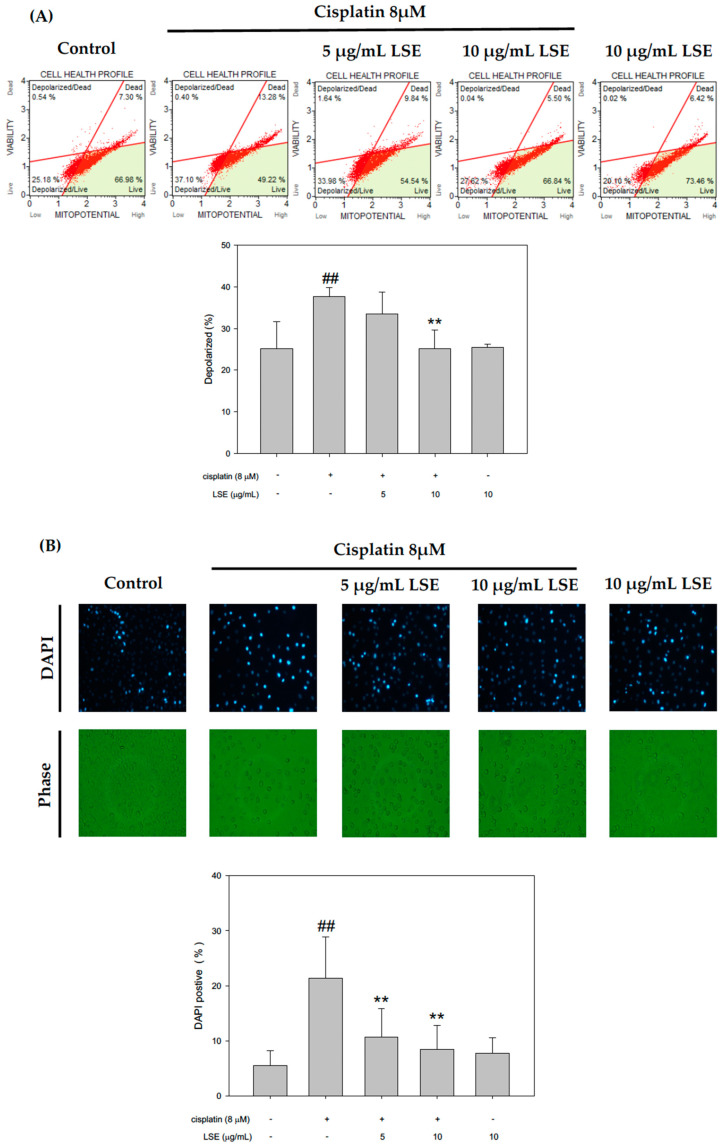

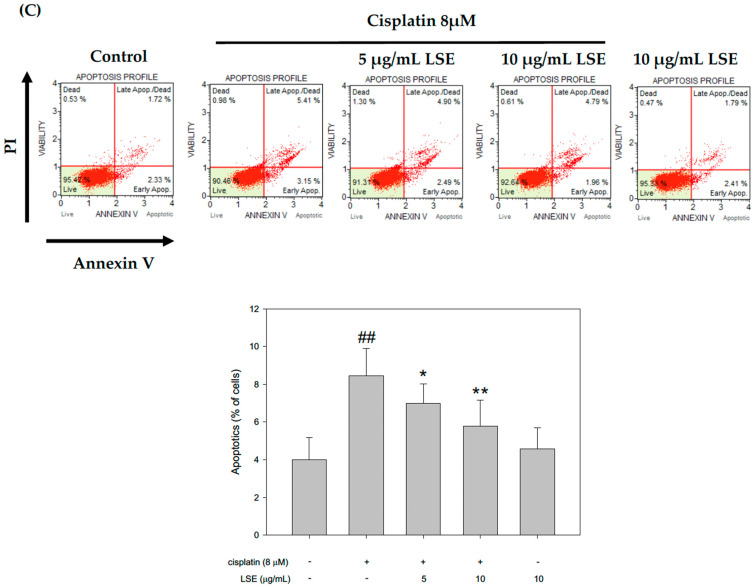
Effect of LSE on cisplatin-induced apoptosis in NRK−52E cells. NRK−52E cells were treated with or without LSE (5, 10 μg/mL) for 24 h in the presence or absence of cisplatin (8 μM). (**A**) The depolarization was assayed by JC-1 staining with flow cytometry. (**B**) The apoptotic cells were assayed by DAPI staining and the DAPI-positive cells were presented as a percentage of the total number of cells. (**C**) The apoptotic cells were analyzed by flow cytometry and apoptotic values were the percentage of apoptotic cells divided by the total number of cells. Quantification data were presented as mean ± SD of three independent experiments by using Sigma plot 10.0 software. ## *p* < 0.01 compared with the control group; * *p* < 0.05 compared with the cisplatin group, ** *p* < 0.01 compared with the cisplatin group.

Apoptosis associated with mitochondrial dysfunction and thus mitochondrial membrane depolarization, was analyzed through a JC-1 staining assay. The results were shown in Figure 3A. Compared with the control group, mitochondrial membrane depolarization in the cisplatin group was significantly increased by 50.2%. After treatment with LSE (5 and 10 μg/mL), mitochondrial membrane depolarization was significantly decreased by 11.1% and 33.27%, respectively. To further investigate whether LSE improves the cisplatin-induced apoptosis in NRK−52E cells, cisplatin-induced apoptosis in NRK−52E cells was analyzed by DAPI staining and Annexin V/PI. As shown in Figure 3B, the fluorescence intensity in the cisplatin group was significantly increased compared to the control group. With co-treatment of LSE (5 and 10 μg/mL) and cisplatin, the fluorescence intensities were significantly reduced compared to the cisplatin group. The results of apoptosis rate in different treatment groups by Annexin V/PI were shown in Figure 3C. It was observed that the apoptosis percentage in the cisplatin group was significantly increased compared to the control group. However, co-treatment of LSE (5 and 10 μg/mL) reduced the apoptosis rate by 17.4% and 31.6% compared with the cisplatin group. The results of DAPI staining appeared in a similar tendency to Annexin V/PI, which indicated that the LSE intervention could prevent cisplatin-induced mitochondrial dysfunction and cell apoptosis.

### 2.4. Effect of LSE on Cisplatin-Induced Apoptotic Pathway in NRK-52E Cells

**Figure 4:**
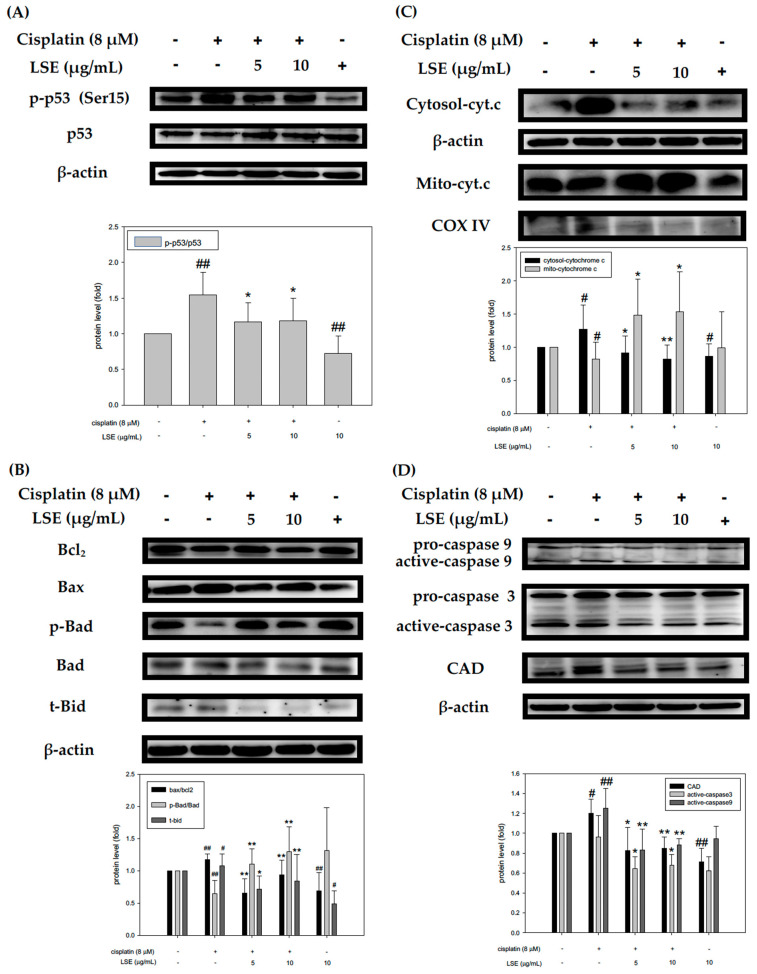
Effect of LSE on cisplatin-induced apoptotic pathway in NRK−52E cells. NRK−52E cells were treated with or without LSE (5, 10 μg/mL) for 24 h in the presence or absence of cisplatin (8 μM). (**A**) The protein levels of p53 and phospho−p53. (**B**) The protein levels of Bcl2, Bax, p−Bad, Bad, and t−Bid. (**C**) The protein levels of cytosol and mitochondrial cytochrome c. (**D**) The protein levels of pro−caspase 9, caspase 9, pro−caspase 3, caspase 3, and CAD were analyzed by Western blotting. Quantification data were presented as mean ± SD of three independent experiments by using Sigma plot 10.0 software. # *p* < 0.05 compared with the control group, ## *p* < 0.01 compared with the control group; * *p* < 0.05, ** *p* < 0.01 compared with the cisplatin group.

According to the above results, the further effect of LSE on the apoptotic-related pathway was investigated. A previous study indicated that DNA damage leads to the activation of p53 for apoptosis [11]. The phosphorylation-p53/p53 level in the cisplatin group was significantly increased by 54.8% compared with the control group, whereas it was significantly reduced in the LSE (5 and 10 μg/mL) and cisplatin co-treatment groups (Figure 4A). Bcl-2 family proteins played a critical role in cell apoptosis. Thus, Bcl−2 family proteins including Bax, Bcl2, Bad and t-Bid were tested. Figure 4B showed that Bax/Bcl2 and t-Bid levels were increased by cisplatin induction compared to the control group. When it comes to the LSE and cisplatin co-treatment groups, the expressions of Bax/Bcl2 and t-Bid were significantly reduced compared to the cisplatin group. p-Bad/Bad levels in the LSE and cisplatin co-treatment groups were significantly increased by 70.5% and 100.4%, respectively. Moreover, mitochondrial depolarization was connected with cytochrome C release [18], and thus, cytochrome C levels in the cytosol and mitochondria were checked. As shown in Figure 4C, the cytochrome C level in the cytosol in cisplatin group was increased compared to the control group. After co-treatment of cisplatin with different concentrations of LSE (5 and 10 μg/mL), cytochrome C levels in mitochondria were retained and reduced the cytochrome C release to the cytosol. In a previous study, cytochrome C was reported to be involved in the activation of the caspase which executed apoptosis [19]. The expression levels of CAD, active-caspase 3, and active-caspase 9 were analyzed. The expression levels of three proteins in the cisplatin group were significantly higher than in the control group whereas reduced in the 5 and 10 μg/mL of LSE group (Figure 4D). Based on the above results, cisplatin not only triggered ROS production but enhanced the expression levels of apoptosis-related proteins. However, LSE intervention could provide protective effects to reduce cisplatin-induced ROS damage and apoptosis in NRK−52E cells.

### 2.5. Effects of LSE on the Kidney Biochemical Parameters, Lipid Peroxidation, and Antioxidant Capacity in Nude Mice Induced by Cisplatin Treatment

**Figure 5:**
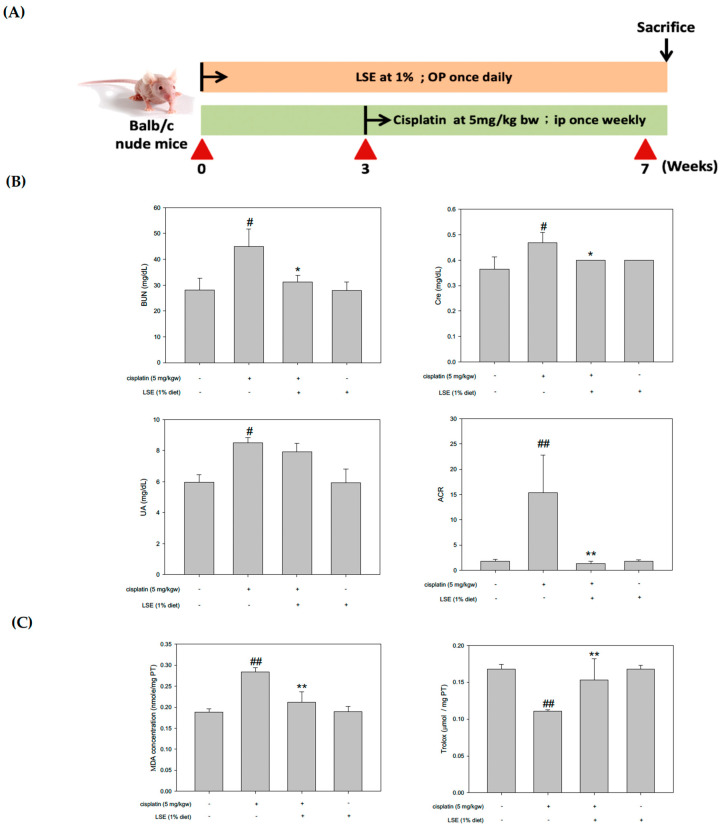
Effects of LSE on the kidney biochemical parameters, lipid peroxidation, and antioxidant capacity in nude mice induced by cisplatin treatment. (**A**) BALB/c nude mice model and workflow (**B**) Serum BUN (Blood urea nitrogen), Cre (creatinine), UA (urea acid) and urine ACR (albumin to creatinine ratio) levels were analyzed. (**C**) The lipid peroxidation of the kidney was assessed by measuring the TBARS assay and the antioxidant capacity of the kidney was assessed by measuring the TEAC assay. Quantification data were presented as mean ± SD of three independent experiments by using Sigma plot 10.0 software. # *p* < 0.05, ## *p* < 0.01 compared with the control group; * *p* < 0.05, ** *p* < 0.01 compared with the cisplatin group.

Due to the nephroprotective effects of LSE in cisplatin-induced NRK−52E cells, a nude mice model was established to understand the in vivo effect of LSE using nude mice by cisplatin induction (Figure 5A). All groups were sacrificed. Kidney tissues and serum were collected for further analysis. The blood urea nitrogen (BUN), serum creatine, uric acid, and urine albumin to creatinine ratio (ACR) levels were shown in Figure 5B. Compared to the control group, the BUN, serum creatine, uric acid, and ACR levels in the cisplatin group were significantly increased. In the 1% LSE group, the BUN, serum creatine, and ACR levels were significantly reduced by 30.5%, 14.5%, and 91.52%, respectively, compared to the cisplatin group. However, uric acid in the 1% LSE group was no different compared to the cisplatin group. As shown in Figure 5C, assessments of kidney lipid peroxidation and antioxidant capacity in cisplatin-induced nude mice were performed. Lipid peroxidation in the 1% LSE group was significantly decreased by 25.4% compared with the cisplatin group, and the antioxidant capacity was significantly increased by 38.1%. These results indicate that LSE could prevent cisplatin-induced renal damage via lower lipid peroxidation and higher antioxidant capacity.

### 2.6. Effects of LSE on Kidney Histopathology and Apoptosis in Nude Mice Induced by Cisplatin Treatment

**Figure 6:**
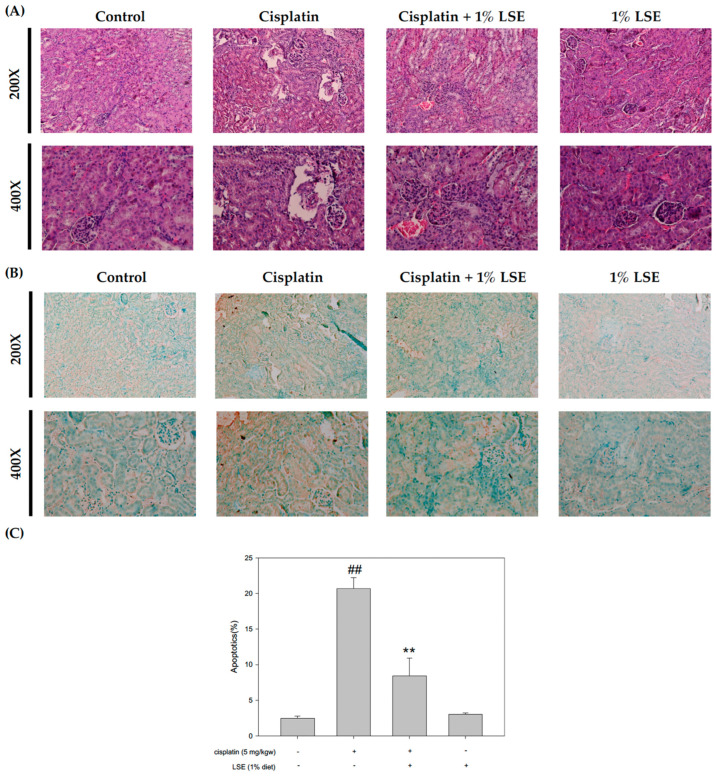
Effects of LSE on kidney histopathology and apoptosis in nude mice induced by cisplatin treatment. (**A**) Representative 200X and 400X images of kidney sections stained with hematoxylin-eosin to display renal structure. (**B**) Representative 200X and 400X images of kidney sections stained with TUNEL to display renal apoptosis degree. (**C**) Quantification data of kidney apoptosis were presented as mean ± SD of three independent experiments by using Sigma plot 10.0 software. ## *p* < 0.01 compared with the control group; ** *p* < 0.01 compared with the cisplatin group.

The kidney histological assessment was applied to determine the protective effect of LSE on cisplatin-induced renal injury. As shown in Figure 6A, cisplatin treatment caused several visible histology changes, including renal tubules with dilation and vacuolization. Although hematoxylin-eosin staining of the kidney sections showed vacuolization in the cisplatin group, this was reduced in the 1% LSE group to a level similar to the control group. To further investigate the effect of LSE on cisplatin-induced apoptosis, the histological section using terminal deoxynucleotidyl transferase-mediated dUTP nick end labeling (TUNEL) was performed. In the microscope, it could be observed that the number of TUNEL-positive cells significantly increased in the cisplatin group. However, the 1% LSE group showed significantly fewer TUNEL-positive cells compared to the cisplatin group (Figure 6B,C). Based on these results, LSE could suppress apoptosis in the kidney after cisplatin induction.

### 2.7. Effect of LSE on Kidney Expression of Apoptosis-Related Proteins in Nude Mice Induced by Cisplatin Treatment

**Figure 7:**
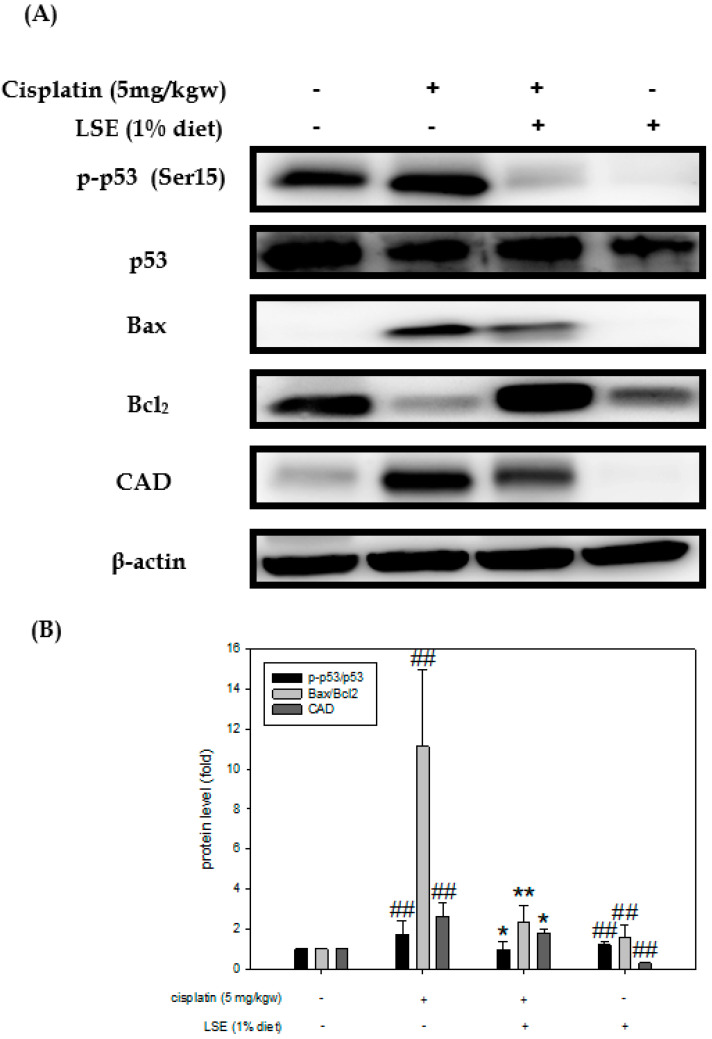
Effect of LSE on kidney expression of apoptosis-related proteins in nude mice induced by cisplatin treatment. (**A**) The protein levels of p53, *p*-p53, Bax, Bcl2, and CAD were analyzed by Western blotting. (**B**) Quantification data were presented as mean ± SD of three independent experiments by using Sigma plot 10.0 software. ## *p* < 0.01 compared with the control group; * *p* < 0.05, ** *p* < 0.01 compared with the cisplatin group.

Mouse kidney tissues were analyzed by Western blotting to investigate the expression of apoptosis-related proteins such as p53, Bax, Bcl2, and CAD. As shown in Figure 7A, p53 phosphorylation was stimulated after cisplatin treatment but suppressed by 1% LSE. Meanwhile, the Bax/Bcl2 and CAD level ratio in the 1% LSE group was significantly decreased by 79.17% compared to the cisplatin group which revealed similar results to the in vitro study (Figure 7B). Both in vitro and in vivo studies indicated that LSE treatment suppressed ROS production and expression of apoptosis-related proteins to protect NRK-52E cells from damage from cisplatin induction.

## 3. Discussion

Cisplatin has been considered one of the most widely used and successful chemotherapy drugs. Until now, chemotherapy resistance and severe side effects are the two major predicaments when it comes to using cisplatin [5]. The main dose-limiting side effect of cisplatin in clinical treatments is nephrotoxicity [20]. Although cisplatin derivatives including carboplatin and oxaliplatin were synthesized to reduce side effects in normal tissues, their therapeutic effects were still limited compared with cisplatin [21]. Thus, it is important to reduce cisplatin-induced nephrotoxicity through the development of natural materials. This study first investigated the protective effect of LSE on cisplatin-induced nephrotoxicity via in vitro and in vivo studies. The results showed that cisplatin led to nephrotoxicity, which was involved in raising the cell death rate, increasing ROS production, and promoting apoptosis in NRK−52E cells. LSE intervention could prevent cisplatin-induced oxidative stress and apoptosis.

An accumulation of ROS levels has been considered the major mechanism in the pathogenesis of cisplatin-induced nephrotoxicity [22,23]. Cisplatin-induced nephrotoxicity can be reduced by inhibiting oxidative stress and apoptosis to cisplatin [24]. In a previous study, several antioxidative agents had been reported to ameliorate cisplatin-induced oxidative stress [25], whereas, cisplatin-induced ROS levels in NRK−52E cells were treated with LSE in the present study. The data exhibited that ROS production in the cisplatin group was significantly increased compared to the the control group, while LSE significantly decreased the cisplatin-induced increase in ROS in NRK−52E cells. However, a previous study has shown that ROS could induce apoptosis via oxidative stress [26]. Cisplatin-induced NRK−52E cells were stained with DAPI and Annexin V/PI staining. The results showed that LSE significantly reduced the oxidative stress and inhibited cisplatin-induced apoptosis in NRK−52E cells (Figure 3).

Some evidence indicates that the accumulation of cisplatin-induced ROS in mitochondria resulted in apoptosis [27,28]. When it comes to increased ROS levels, a series of signaling cascades such as p53 would be activated to damage DNA [29]. Cisplatin interfered with the balance of anti- and proapoptotic proteins, which reduced the expression of Bcl2 and induced translocation of Bax to accelerate cytochrome c release and caspase-9 activation [30]. Thus, to further verify the mechanisms of LSE on cisplatin-induced apoptosis in NRK−52E cells, co-treatment of LSE with cisplatin was analyzed. This study revealed that LSE could inhibit cisplatin-induced apoptosis through down-regulating p53, Bax, and t-Bid expression and up-regulating Bcl2 and p-Bad expression (Figure 4A and 4B). Meanwhile, mitochondria membrane depolarization and the cytochrome c level were increased in cisplatin group (Figure 4C). Once mitochondria membrane permeability was destroyed, cytochrome c would be released into the cytoplasm [31]. However, LSE treatment not only reduced the mitochondria membrane depolarization but also restored cytochrome c in mitochondria. Besides, the expression levels of active caspase 3 and 9, which demonstrated that LSE exerted a protective effect on cisplatin-induced apoptosis in NRK−52E cells (Figure 4D). The above results indicated that LSE inhibited the intrinsic apoptosis pathway to protect cisplatin-induced NRK−52E cells.

To comprehend and confirm the protective effects of LSE in vivo, a cisplatin-induced BALB/c nude mice model was used. The pathogenesis of cisplatin-induced nephrotoxicity not only involved renal structure changes but also resulted in oxidative stress and apoptosis. It has been reported that cisplatin leads to lipid peroxidation in the kidneys by increasing ROS production. Meanwhile, lipid peroxidation is connected with an increase in serum urea acid and serum creatinine levels in the kidneys [32]. The albumin to creatinine ratio (ACR) is commonly used for identifying kidney disease [33]. Consistent with the previous study, the data of in vivo experiments exhibited cisplatin-induced nephrotoxicity, while LSE treatment could decrease the serum urea acid, BUN, serum creatinine and ACR ratio (Figure 5B). Furthermore, lipid peroxidation was also reduced and TEAC was improved after LSE treatment (Figure 5C). Cisplatin-induced nephropathy could be observed several changes including degeneration, vacuolization and apoptosis in the proximal tubule cells [34,35]. Consistent with these studies, the histological results of LSE treatment in this study showed that cisplatin-induced the nephropathy of nude mice, whereas LSE treatment could prevent nephrotoxicity induced by cisplatin. TUNEL staining is frequently considered a suitable marker for apoptotic cells. While the apoptotic cells are in progress, TUNEL staining would be positive [36]. In this study, administration of cisplatin induction resulted in positive TUNEL staining in the kidneys of nude mice; however, the number of TUNEL-positive cells in the LSE treatment group was significantly reduced indicating reduced apoptosis in this group. Based on these results, the molecular mechanism of LSE’s protective effect on cisplatin-induced apoptosis in vivo was analyzed. Consistent with our in vitro study, the expressions of pro-apoptotic proteins (Bax and CAD) and the phosphorylation of p53 were downregulated by LSE treatment, while the expression of anti-apoptotic proteins (Bcl2) was up-regulated. According to the findings of these in vitro and in vivo studies, we suggested that LSE could improve cisplatin-induced apoptosis in vitro and in vivo for the following perspectives: (i) reduction in ROS levels; (ii) down-regulation of p53 phosphorylation; and (iii) inhibition of pro-apoptotic proteins and caspase 3 and 9 expressions.

Q3G, known as quercetin derivative, plays a critical role in antioxidation and is one of the most abundant flavonoids in plant foods [37]. Q3G is absorbed more easily than the aglycone forms [38,39]. It was reported that Q3G could reduce ROS production to prevent alcohol-induced cytotoxicity [40] and improve insulin resistance by modulating IRS-1 [41]. In a previous study, the bioactive compounds in LSE were analyzed by HPLC/ESI-MS-MS and were found to be Q3G [13]. Consistent with this previous study, the results of in vitro and in vivo experiments in the present study showed that LSE rich in Q3G alleviated cisplatin-induced nephrotoxicity by modulating ROS production and apoptosis pathways. Currently, the data and above information demonstrate that the nephroprotective effects of LSE may be based on its bioactive compound (Q3G).

The strengths of this present study showed the original investigation of LSE on cisplatin-induced nephrotoxicity. These findings provide a new perspective on the prevention of cisplatin-induced renal damage. However, the limitations are that the effect of LSE on cisplatin-induced inflammatory cytokine expression and the related pathway are still unknown, which need to explored in the future.

## 4. Materials and Methods

### 4.1. Chemical and Reagents

The cisplatin used in the present study was obtained from Acros Organics (Geel, Belgium). Dulbecco’s Modified Eagle’s Medium (DMEM), fetal bovine serum and penicillin/streptomycin were purchased from Hyclone Laboratories (Logan, UT, USA). The primary antibodies including Bax, Bcl2, Bad, CAD, caspase 3, caspase 9, cytochrome C, COX-IV, p53, p-Bad, and t-Bid, were purchased from Santa Cruz (Dallas city, TX, USA). β-actin was purchased from Sigma-Aldrich (St. Louis, MO, USA). Phospho-p53 was purchased from Cell Signaling Technology (Beverly, MA, USA). The secondary antibody conjugated horseradish peroxidase was purchased from Sigma-Aldrich (St. Louis, MO, USA).

### 4.2. Lotus Seedpod Extract (LSE) Preparation

Lotus seedpods (cultivar: sheklian) were extracted following the published procedure [13]. In brief, dried lotus seedpods (150 g) were boiled in 6 L deionized water at 95 °C for 2 h. After cooling to room temperature, the residues of lotus seedpod were filtered through Grade No. 1 filter paper (Whatman, Maidstone, UK) and then the crude extract was lyophilized under vacuum at −85 °C as lotus seedpod extract (LSE) powder. LSE powder was stored at 4 °C before use.

### 4.3. Cell Culture

NRK−52E rat kidney proximal tubular epithelial cells were obtained from Bioresource Collection and Research Center (Food Industry Research and Development Institute, Hsinchu City, Taiwan). NRK-52E cells were cultured in DMEM supplemented with 5% FBS, and 1% penicillin/streptomycin at 37 °C in a 5% CO_2_ incubator.

### 4.4. Cell Viability Assay

NRK−52E cells were seeded in a 6−well dish (2 × 10^5^ cells/dish) for 24 h. The cells were treated with/without LSE (5, 10, 25, 50 and 100 μg/mL) or cisplatin (2, 4, 6, 8 and 10 μM) for 24 h. Cell viability assay was analyzed with podium iodine (1 mg/mL) by Guava^®^ Muse^®^ cell analyzer (Luminex, Austin, TX, USA).

### 4.5. DAPI Stain Assay

NRK−52E cells were seeded in a 6−well dish (2x10^5^ cells/dish) for 24 h. NRK−52E cells were treated with or without LSE (5 and 10 μg/mL) for 24 h in the presence or absence of cisplatin (8 μM). After 24 h, the medium was removed and 4% formalin was added to fix for 30 min. Next, DAPI (Sigma-Aldrich, St. Louis, MO, USA) was diluted with PBS and the nucleus was stained for 20 min at room temperature in the dark. Cell morphology was captured by fluorescence microscopy (Bio-Rad, Hercules, CA, USA) and quantified by ImageJ.

### 4.6. Annexin V/PI Stain Assay

Annexin V/PI Stain assay was executed using an Annexin V: PE apoptosis detection kit (#559763, BD sciences, San Jose, CA, USA). NRK−52E cells were resuspended with DMEM and stained with 0.5 μL Annexin V-PE and 1 μL 7-AAD at room temperature for 15 min in the dark. Cell apoptosis was analyzed by Guava^®^ Muse^®^ cell analyzer (Luminex, Austin, TX, USA).

### 4.7. JC-1 Stain Assay

JC-1 is a lipophilic, cationic dye that is able to enter into the mitochondria where it accumulates due to changes in the mitochondrial membrane potential. Briefly, the medium was removed and NRK−52E cells were rinsed with PBS. Next, JC-1 was mixed with DMEM and added to NRK−52E cells for 30 min in the dark. After incubation, cells were washed with PBS and analyzed by Guava^®^ Muse^®^ cell analyzer (Luminex, Austin, TX, USA).

### 4.8. Reactive Oxygen Species (ROS) Analysis

ROS from cisplatin-induced NRK−52E cells were evaluated using Muse^®^ oxidative stress kit. According to the manufacturer’s protocol, NRK−52E cells were prepared in assay buffer at 1 × 10^6^ cells/mL and then oxidative stress reagent was diluted in assay buffer. An amount of 190 μL of oxidative stress working reagent was added to 10 μL of cells. After incubation at 37 °C for 30 min, production of ROS from cisplatin-induced NRK−52E cells was analyzed by Guava^®^ Muse^®^ cell analyzer (Luminex, Austin, TX, USA).

### 4.9. Mitochondria Isolation

Mitochondria from NRK−52E cells were isolated using a Thermo Mitochondria isolation Kit (Rockford, IL, USA). According to the manufacturer’s protocol, NRK−52E cells were harvested, added with reagent into the Eppendorf tube, vortexed for 2 h on ice, and then protein inhibitor (Na_3_VO_4_ and PMSF) was added into the Eppendorf tube. After centrifuging twice, the pellet containing mitochondria was resuspended. Mitochondrial concentration was determined by a commercial BCA kit (Energenesis Biomedical Co., LTD, Taipei City, Taiwan).

### 4.10. Animal and Experimental Design

Six-week-old male BALB/c nude mice were purchased from BioLASCO Taiwan Co., Ltd. (Taipei City, Taiwan). Mice were housed in a room with constant conditions of temperature (22 ± 2 °C) and humidity (55 ± 2%) on a 12-h light/dark cycle. After adaptation for a week, mice were randomly divided into the following four groups (7 mice per group): control, cisplatin, cisplatin + LSE (1% added in diet), and LSE (1% added in diet). LSE was mixed with a normal diet before use. BALB/c nu/nu mice were pre-treated with LSE (1%, op. once daily) for 3 weeks, and then treated with cisplatin (5 mg/kg BW, ip. once weekly) with or without LSE (1%, op. once daily) for 4 weeks. After 7 weeks, all mice were sacrificed. Kidney tissue and serum were collected for analysis.

### 4.11. Urine and Serum Biochemical Parameters

The urine sample was collected from metabolic cages before sacrifice. The serum sample was collected using EDTA tubes and centrifuged at 1310× *g* for 15 min at 4 °C to determine serum biochemistry parameters. Serum BUN (blood urea nitrogen), creatinine, urea acid and urine ACR (albumin to creatinine ratio) levels were determined by the medical laboratory at Chung Shan Medical University Hospital.

### 4.12. Thiobarbituric Acid Reactive Substances (TBARS) Assay

The lipid peroxidation level was measured by TBARS. TBARS assay was executed following the published procedure [42]. TBARS was determined by reading 532 nm using a spectrophotometer and by comparison with a standard curve of malondialdehyde (MDA).

### 4.13. Trolox Equivalent Antioxidant Capacity (TEAC) Assay

The TEAC assay was described in a previous study [43]. In brief, the ABTS^+^ stock solution was composed of 500 µL peroxidase, 500 µL ABTS solution, and 500 µM hydrogen peroxide and then stored in the dark. The diluted sample was mixed with 500 µL of ABTS, and the absorbance of the mixture was measured at 734 nm after 6 min. Trolox was standard for TEAC assay.

### 4.14. Hematoxylin-Eosin Staining

The kidney was collected and fixed in 10% buffered formalin. After fixation, the tissue was dehydrated with ethanol and xylene, embedded in paraffin, and then cut into about 5 μm thick sections, stained with hematoxylin-eosin.

### 4.15. TUNEL Assay

The TUNEL assay was performed using Apo-BrdU-IHCTM In Situ DNA Fragmentation Assay Kit (BioVision, Mountain View, CA, USA) according to the manufacturer’s instructions. Briefly, kidney sections were covered with proteinase K for 20 min, followed by 3% H_2_O_2_ in methanol for 5 min, to inactivate endogenous peroxidase activity. The sections were rinsed with PBS and covered with a blocking solution to reduce background staining. The sections were then incubated with avidin−peroxidase complexes in PBS for 30 min and rinsed with PBS for 5 min. A dark brown color, indicating DNA breaks, developed after incubation with DAB (3,3′-diaminobenzidine) solution, followed by counterstaining with methyl green solution.

### 4.16. Protein Extraction and Western Blotting

Proteins from NRK-52E cells and mice kidney tissue were homogenized by RIPA lysis buffer containing protein inhibitor and centrifuged at 12,000× *g* for 15 min at 4 °C. The supernatant was stored at −80 °C until use. The protein concentrations of cell lysates and kidney tissue were determined by a commercial BCA kit (Energenesis Biomedical Co., LTD, Taipei City, Taiwan). A fixed amount (20-50 μg/lane) of protein was loaded and separated by 8-15% SDS-PAGE. Proteins were transferred to the PVDF membrane (Merk, Darmstadt, Germany) and the membrane was blocked by 5% non-fat dry milk at 4 °C for 1 h. After blocking, the membrane was incubated with primary antibody overnight at 4 °C. After washing three times by TBS with Tween 20 (TBST), the horseradish peroxidase-conjugated secondary antibody was applied to the membrane at 4 °C for 1 h. After washing three times by TBST, visualization of protein bands was performed using an enhanced chemiluminescence reagent. β-actin was used as internal control and protein levels were quantified by ImageQuantTM LAS 4000 mini (GE Healthcare Bio-Science AB, Uppsala, Sweden).

### 4.17. Statistical Analysis

Sigma Plot 10.0 (Systat Software, Inc., San Jose, CA, USA) software was used for statistical analysis. Data were reported as the mean ±standard deviation (SD). Statistical significance was analyzed by Student’s *t*-test. Significant differences were established at *p* < 0.05.

## 5. Conclusions

LSE raised cisplatin-induced cell viabilities, had lower ROS production, and reduced the expression of apoptosis-related proteins in NRK−52E cells. Simultaneously, histopathological changes and the apoptosis−inhibiting mechanism of LSE in cisplatin-induced nude mice were revealed. LSE exerted nephroprotective effects against cisplatin, whether in vitro or in vivo, and this was the first study to display the possible mechanism of LSE on cisplatin-induced nephrotoxicity. LSE could be a novel and natural chemoprotective agent for cisplatin chemotherapy in the future.

## Data Availability

Data are available on request to the corresponding author.
